# Formation and Validation of the Healthy Diet Index (HDI) for Evaluation of Diet Quality in Healthcare

**DOI:** 10.3390/ijerph18052362

**Published:** 2021-02-28

**Authors:** Jaana Lindström, Kirsikka Aittola, Auli Pölönen, Katri Hemiö, Kirsti Ahonen, Leila Karhunen, Reija Männikkö, Ulla Siljamäki-Ojansuu, Tanja Tilles-Tirkkonen, Eeva Virtanen, Jussi Pihlajamäki, Ursula Schwab

**Affiliations:** 1 Department of Public Health and Welfare, Finnish Institute for Health and Welfare, 00271 Helsinki, Finland; Katri.hemio@thl.fi (K.H.); Eeva.virtanen@thl.fi (E.V.); 2 School of Medicine, Institute of Public Health and Clinical Nutrition, University of Eastern Finland, 70211 Kuopio, Finland; Kirsikka.aittola@uef.fi (K.A.); Leila.karhunen@uef.fi (L.K.); Reija.mannikko@terveystalo.com (R.M.); Tanja.tilles-tirkkonen@uef.fi (T.T.-T.); Jussi.pihlajamaki@uef.fi (J.P.); Ursula.schwab@uef.fi (U.S.); 3 Division 1, Tampere University Hospital, 33520 Tampere, Finland; auli.polonen@pshp.fi; 4 Clinical Nutrition Unit, Tampere University Hospital, 33520 Tampere, Finland; kirsti.ahonen617@gmail.com (K.A.); Ulla.siljamaki-ojansuu@pshp.fi (U.S.-O.); 5 Department of Medicine, Endocrinology and Clinical Nutrition, Kuopio University Hospital, 70029 Kuopio, Finland

**Keywords:** chronic diseases, diet quality, evaluation, prevention, type 2 diabetes, validation

## Abstract

Lack of tools to evaluate the quality of diet impedes dietary counselling in healthcare. We constructed a scoring for a validated food intake questionnaire, to measure the adherence to a healthy diet that prevents type 2 diabetes (T2D). The Healthy Diet Index (HDI) consists of seven weighted domains (meal pattern, grains, fruit and vegetables, fats, fish and meat, dairy, snacks and treats). We studied the correlations of the HDI with nutrient intakes calculated from 7-day food records among 52 men and 25 women, and associations of HDI with biomarkers and anthropometrics among 645 men and 2455 women. The HDI correlated inversely with total fat (Pearson’s *r* = −0.37), saturated fat (*r* = −0.37), monounsaturated fat (*r* = −0.37), and the glycaemic index of diet (*r* = −0.32) and positively with carbohydrates (*r* = 0.23), protein (*r* = 0.25), fibre (*r* = 0.66), magnesium (*r* = 0.26), iron (*r* = 0.25), and vitamin D (*r* = 0.27), (*p* < 0.05 for all). In the linear regression model adjusted for BMI and age, HDI is associated inversely with waist circumference, concentrations of fasting and 2-h glucose and triglycerides in men and women, total and LDL cholesterol in women, and fasting insulin in men (*p* < 0.05 for all). The HDI proved to be a valid tool to measure adherence to a health-promoting diet and to support individualised dietary counselling.

## 1. Introduction

An unhealthy diet is acknowledged as one of the leading contributors to many chronic diseases [1]. For example, lifestyle counselling provided to individuals with increased risk for type 2 diabetes (T2D) in accordance with the general dietary recommendations has been shown to improve risk factors and reduce T2D incidence [2]. Hence, dietary counselling should be one of the cornerstones of the prevention and management of T2D and several other chronic diseases [3]. 

In the healthcare setting, dietary counselling is often provided by professionals without specific training in nutrition, and one of the identified barriers is the lack of easy and concrete tools for diet evaluation and feedback [4,5]. Such a tool should be easy to comprehend, focused on the behaviours that are relevant to the risk, and accommodating to the person’s risk factors and present diet [4,6,7]. Furthermore, the tool should facilitate the use of behaviour change techniques such as monitoring and feedback on dietary intake that are recommended to enhance the effectiveness of counselling [8]. 

In the research setting, nutrient intakes are typically assessed rigorously [9] with laborious methods such as food records and comprehensive food frequency questionnaires. Many available diet quality scores are based on these rigorous methods and often apply dichotomised, yes/no-type cut-off points for dietary goals [10]. However, in healthcare practice, easier and quicker methods that enable immediate feedback and advice without nutrition professionals’ input would be more feasible [11]. Furthermore, acknowledging also smaller changes in the preferred direction could be a motivational factor for the individual [12]. 

Lassale et al. [13] assessed the performance of ten validated diet quality scores in predicting mortality and found an inverse association between all tested scores and mortality. However, they noticed a clear variation in the scores and their performance in different populations. Aljuraiban et al. [14] reviewed 31 diet quality scores related to cardiovascular disease risk and they also emphasize the importance of accounting for cultural differences when applying a score. 

Finnish examples of T2D prevention implementation programs with a strong focus on lifestyle intervention include the FIN-D2D (2003–2008) conducted as part of the National diabetes program DEHKO [15], the Finnish Airline diabetes prevention project (2006–2010) [16], and Stop Diabetes (StopDia, 2016–2019) [17]. In the FIN-D2D, a 16-item Food Intake Questionnaire (D2D-FIQ) was created, to be a tool to facilitate dietary counselling conducted by public health nurses. Later, the D2D-FIQ was scientifically validated in the Airline diabetes prevention project and proven to be a reliable and reasonably accurate method to estimate dietary intake [18]. Finally, a slightly updated version of the D2D-FIQ was used also in the StopDia [17] as a tool to collect data on participants’ habitual diet and to measure their adherence to the diet to prevent T2D.

The aim of the present study was to construct a Healthy Diet Index (HDI), derived from the D2D-FIQ [18], and to study its criterion validity. Ultimately, we aimed to create a tool for the evaluation of diet as compared to dietary recommendations, for the assessment of the compliance with dietary intervention to prevent T2D and other chronic diseases, and for supporting individualised dietary counselling in healthcare. 

## 2. Materials and Methods

### 2.1. Update of the D2D-FIQ and Development of the Scoring

The original 16-item D2D-FIQ was compiled to cover the most important dietary behaviours in the development of T2D [18]. Based on user experience and accumulating research, we updated the 16-item version to an 18-item version (see Appendix A), to include two separate questions on the consumption of main meals (breakfast; lunch; dinner) and snacks (morning snack; afternoon snack; evening snack; other snacks) consumed during a regular workday instead of the single question “how many meals and snacks do you consume on a typical day” in the 16-item version. Additionally, a new question about the consumption of nuts, seeds, and almonds was included and for the question on vegetable consumption, a category of “≥3 servings per day” was added. Furthermore, some food item examples that were listed in the questionnaire were updated to comply with the supply. 

In order to ensure the construct and content validity of the tool, three groups of experienced nutritionists were established in Helsinki (Finnish Institute for Health and Welfare, *n* = 5), Kuopio (University of Eastern Finland, *n* = 5), and Tampere (Tampere University Hospital and Pirkanmaa Hospital District, *n* = 6). Following a modified Delphi method [19], each group member was first asked to individually study the D2D-FIQ and reflect it in the context of the Finnish national nutrition recommendations published in 2014 [20] as well as research evidence specifically related to dietary risk and protecting factors of T2D [21,22,23,24,25,26,27]. After that, the groups gathered separately, multiple times if needed, to discuss the relative importance of the questions as regards T2D and healthy diet in general, and to construct tentative scoring for the D2D-FIQ questions’ answers. Finally, joint discussions of all groups were arranged, in order to reach a consensus between the experts and to finalise the scoring.

A set of domains was agreed upon, to give an overview of characteristics of a health-promoting diet. The points for each domain were assigned to reflect their estimated relative importance in a healthy, T2D preventing diet so that the “optimal” consumption pattern yields maximum score and deviation to either direction yields lower points. Finally, the Healthy Diet Index (HDI) was constructed (see Appendix B, Table A1). It contains seven main domains, weighted depending on their agreed relative importance in a diet to prevent T2D. Within each domain, increasing points indicated a more healthy diet. The maximum total HDI was set at 100 points. 

The justification for the HDI domains, as well as the scoring in detail is presented in Appendix B, Table A1. 

The domain “meal pattern” (0–10 points) was defined based on the number and timing of eating occasions during a typical weekday, as well as the number of main dishes (as an indicator of a cooked meal rather than a snack-type meal) during a week. Maximum points were given for a pattern including a meal and/or a snack in the morning, in the mid-day, and in the evening, having altogether 4–6 eating occasions during a weekday, and at least 7 main dishes during a week. While the research evidence to recommend a set meal pattern is not very strong [28,29] some studies have shown that breakfast skipping is related to increased risk of dysglycaemia and T2D [25] and obesity in the long run [30].

Based on the strong evidence [23] linking whole grains and T2D, a high weight was given on domain “grains” (0–20 points). The consumption was graded based on fibre content, with 6 or more “fibre-adjusted cereal units” plus low consumption (one portion per day or less) of low-fibre cereal products such as white bread or breakfast cereal. 

Also, the domain “fruit and vegetables” (0–20 points) was considered important, with 3 daily servings of vegetables and 2 daily servings of fruit yielding maximum points. Fruit and vegetables have an acknowledged role in the health-promoting diet in general [31,32] and their association with T2D risk is supported by evidence from both observational [23] and intervention studies [33,34]. 

The domain “fats” (0–15 points) covers the quality of fat used for cooking, in salad dressing, and as a spread on bread, as well as the fat from nuts and seeds, with higher points given for favouring foods that are a source of unsaturated fat in the diet. The relatively high weight was based on the evidence on the association of fat quality with T2D [35,36] and cardiovascular health [37].

The domain “fish and meat” was scored based on the questions on number of main dishes consumed per week and amount of processed meat consumed per day. Fish and vegetarian dishes were deemed recommendable and meat and sausage dishes to be consumed only in moderation. The epidemiological evidence linking high processed and red meat consumption with T2D is consistent [24] and there is some research suggesting a benefit of fish [23]. 

The scoring for domain “dairy” (0–10 points) was justified by research evidence [27,38] and followed the national recommendations on milk products consumption [20], with the highest score given fofr a consumption pattern of 5–6 dL of low-fat liquid dairy combined with moderate consumption (2–3 slices per day) of reduced-fat cheese.

The domain “snacks and treats” (0–15 points) was constructed to cover several questions on fast foods, salty and sugary snacks, sweets and desserts, and beverages that contain either sugar or alcohol. These food items are generally recommended to be consumed in moderation, if at all [39]. One glass of fruit juice per day was considered to be in line with national recommendations [20] but otherwise, for the items in this domain, non-consumption yielded the highest points. Especially consumption of sugar-sweetened beverages has been shown to associate with increased T2D risk [40] and high consumption of snack-type foods, typically high in sodium, sugar, and/or fat, with lower quality diet [41]. The reasoning for including the alcoholic beverages into this domain was to make visible that they contain a lot of energy but minimally any other nutrients and may thus contribute to the development of obesity [42]. 

### 2.2. Validation Datasets

The external validation dataset was used to assess the performance of the HDI using a 7-day food record as the reference method. The dataset originated from the Finnish Airline diabetes prevention project [16] (“Airline data”) and had been used in the D2D-FIQ validation study [18]. The D2D-FIQ validation study was approved by the Coordinating Ethics Committee of Hospital District of Helsinki and Uusimaa and an informed consent form was signed by all participants, who were volunteers from the Finnish Airline diabetes prevention study. The participants (52 men and 25 women) were advised to think about the past month while answering the D2D-FIQ. Starting from the next day after the D2D-FIQ was completed, the participants were asked to fill in a 7-day food record, using a picture booklet [43] and mechanical scale to estimate portion sizes. The participants were advised to write down in detail all the foods and beverages they consumed. The food records were returned by mail and checked by the study nutritionist (K.H.), who also contacted the participants by phone, when clarification was needed. The nutrient intakes were calculated from the 7-day food records using the in-house software based on the Finnish food composition database [44]. Energy proportions were calculated by multiplying fat grams by 37, carbohydrate, sucrose, and protein grams by 17 and alcohol grams by 29. The energy from each macronutrient was divided by the total energy (kJ) and multiplied by 100 for the final energy percentage (E%). Energy-adjusted fibre intake was calculated per 4186 kJ (equivalent to 1000 kilocalories) by dividing fibre grams by total energy and multiplying by 4186. The HDI was calculated based on the D2D-FIQ. The original D2D-FIQ version used in this study included only 16 items. Therefore the “meal pattern” domain of the HDI was calculated based on a combined question on the number of eating occasions (instead of separate questions on meals and snacks) habitually consumed during a day (see Appendix B, Table A1 for scoring). Furthermore, it was lacking the question about the consumption of nuts, seeds, and almonds, and the maximum for domain “fat” was thus 13 points and consequently, the maximum HDI was 98. 

The cross-sectional baseline dataset from the StopDia diabetes prevention trial [17] (“StopDia data”) was used to evaluate the association of the HDI with clinical risk factors. The study procedures were explained in detail earlier [17]. Briefly, 645 men and 2455 women with increased T2D risk (based on FINDRISC risk score [45], history of gestational diabetes, or impaired fasting glucose or impaired glucose tolerance detected earlier) were recruited into the StopDia randomised controlled trial and filled in comprehensive digital questionnaires, including the updated D2D-FIQ (Appendix A). Based on the D2D-FIQ, the HDI was computed for each participant. Clinical measurements including height (without shoes), weight (in light indoor clothing), waist circumference (2 measurements, midpoint between the lowest ribs and iliac bone, blood pressure (2 measurements with automatic device) were conducted. Fasting blood samples were collected and analysed for HbA1c, concentrations of plasma glucose, total, LDL, and HDL cholesterol, and triglycerides, in the designated laboratories that provided the analyses for the primary healthcare services in the area, and for insulin in the laboratory of the University of Eastern Finland in Kuopio. Furthermore, a two-hour oral 75 g-glucose tolerance test was conducted, with measurement of plasma glucose concentration two hours after consuming the glucose load.

### 2.3. Statistical Analyses

The concurrent criterion validity of the HDI was tested by analysing associations between the HDI (total index and domains) and nutrient intakes calculated from the food records (the Airline dataset) using Pearson’s correlation. Correlation coefficients (modulus) between 0.20 and 0.49 were interpreted to suggest acceptable and 0.50 or more good association [46].

The associations between the HDI and blood biomarkers, anthropometrics and blood pressure (the StopDia dataset) were studied by analysing the trends in adjusted variable means between quarters of the HDI with linear regression. These analyses were conducted separately for men and women, with adjustment for body mass index (BMI) calculated by dividing weight (kg) by height (m) squared, and age when appropriate. Participants with missing information on anthropometric or clinical variables were excluded from the respective analysis. Statistical analyses were performed with SAS version 9.3. 

## 3. Results

The characteristics of the participants in both datasets, including dietary intakes calculated from the 7-day food records in the airline dataset are shown in Table 1.

The mean (±std) HDI within the Airline data was 55 ± 10 (range 40–82) among men and 61 ± 9 (range 44–73) among women, maximum index being 98 (*p* = 0.0149 for difference between men and women). In the StopDia data, the corresponding figures were 59 ± 10 (range 30–94) among men and 63 ± 11 (range 11–93) among women, maximum index being 100 (*p* < 0.001 for difference between sexes).

### 3.1. HDI and Dietary Intake

In the airline dataset (Table 2), the HDI correlated positively with energy proportion of carbohydrates (Pearson’s *r* = 0.23, *p* = 0.0485) and protein (*r* = 0.25, *p* = 0.033), energy-adjusted intake of fibre (*r* = 0.67, *p* < 0.001), magnesium (*r* = 0.25, *p* = 0.024), iron (*r* = 0.25, *p* = 0.029), and vitamin D (*r* = 0.27, *p* = 0.018) calculated from the 7-day food records. An inverse correlation was observed for energy proportion of fat (Pearson’s *r* = −0.37, *p* = 0.001), saturated fat (*r* = −0.39, *p* < 0.001) and monounsaturated fat (*r* = −0.37, *p* = 0.001), and glycaemic index of the diet (*r* = −0.32, *p* = 0.006). All the observed correlations exceeded the threshold of “acceptable” association (|r| ≥ 0.20) between the two measures.

No correlation between the HDI and total energy intake was evident. Many expected correlations were detected also between the HDI domains and nutrient intakes. For example, energy-adjusted fibre intake was positively correlated with points for “grains” and “fruit and vegetables” and also with “fish and meat”, and “snacks and treats”. Energy proportion of saturated fat was inversely correlated with points for “fruit and vegetables” and “dairy” (but not with points for “fats”).

### 3.2. HDI and Clinical Factors

In the StopDia data, HDI was positively correlated with age (*r* = 0.30, *p* < 0.001 and *r* = 0.34, *p* < 0.001) and inversely with BMI (*r* = −0.13, *p* < 0.001 and *r* = −0.15, *p* < 0.001) among women and men, respectively. In Table 3 (women) and Table 4 (men), the blood biomarkers, anthropometrics, and blood pressure are presented by quarters of the HDI. After adjustment for age and BMI, the HDI was inversely associated with waist circumference, fasting plasma glucose, 2-h post-load plasma glucose, and plasma triglycerides in both men and women, respectively. Among women, the HDI was inversely associated also with concentrations of total cholesterol and LDL-cholesterol, and among men, with fasting plasma insulin concentration.

Based on the linear regression model adjusted for age and BMI, it was estimated that for each 10-point increase in HDI, waist circumference decreased by 0.6 cm (*p* < 0.001) and 0.6 cm (*p* = 0.004), fasting glucose concentration by 0.03 mmol/L (*p* = 0.018) and 0.09 mmol/L (*p* = 0.005), 2-h post load glucose concentration by 0.12 mmol/L (*p* = 0.005) and 0.36 mmol/L (*p* < 0.001), and concentration of plasma triglycerides by 0.03 mmol/L (*p* = 0.023) and 0.17 mmol/L (*p* < 0.001), among women and men, respectively.

## 4. Discussion

### 4.1. Summary of the Findings

In this paper, we present the construction and validation of the HDI Healthy Diet Index, which was derived based on the previously validated D2D-FIQ food intake questionnaire [18]. The D2D-FIQ was originally developed to facilitate dietary counselling in the T2D prevention program FIN-D2D in primary healthcare [15]. While developing the HDI our guiding idea was to create an index that depicts the quality of the diet as a whole, makes use of all relevant information available in the D2D-FIQ and is sensitive for beneficial changes in diet even when the “optimal” consumption level is not achieved. 

The HDI and its domains were shown to correlate with nutrient intakes calculated using 7-day food records, the “golden standard” in nutritional epidemiology [9], as a reference method. The correlation coefficients suggested acceptable associations between HDI and energy proportion of carbohydrates, protein, fat and saturated fat as well as magnesium, iron, and vitamin D, and a good association between HDI and fibre density of the diet [46].

Furthermore, the HDI showed expected relationships with clinical and anthropometric risk factors for T2D and cardiovascular diseases in the large StopDia baseline data. Based on these analyses, the criterion validity of the HDI was fulfilled.

### 4.2. Development and Content of the Scoring

Our group included nutrition professionals from several fields and with diverse work experience, which ensured that the *a priori* defined scoring of the HDI was both evidence-based and practice-oriented. Specifically, the landmark Finnish Diabetes Prevention Study (DPS) demonstrating that T2D is preventable by lifestyle intervention [47] was referred to. In the DPS, the adherence to the dietary goals (total fat <30 E%, saturated fat <10 E%, and dietary fibre intake ≥15 g/1000 kcal) was assessed by collecting 3-day food records and by calculating the nutrient intakes with a dietary analysis program [47] and the achievement of the intervention goals was strongly associated with T2D risk reduction [48]. In practice, the DPS participants were encouraged to consume whole grains, fruit and vegetables, low-fat dairy, and vegetable oils and soft margarines, at the expense of nutritionally poor, energy-dense foods such as sweet snacks, low-fibre bread and cereals, and sugar-containing and alcoholic beverages [34]. The approach to base the HDI on the validated D2D-FIQ was practical but also supported by the common-sense fact that as people consume foods and beverages, not nutrients, also dietary counselling should be focused on foods. Furthermore, there is solid epidemiological evidence on the associations between certain food groups and T2D risk [23]. 

In the review and meta-analysis by Schwingshackl et al. [23], observational epidemiological evidence was collated and “optimal” intakes were calculated for the food groups that were deemed either protective against or predisposing to T2D. They suggest that consumption of two daily servings of whole grains, two to three servings of vegetables, two to three servings of fruit, three servings of dairy and non-consumption of red and processed meat, eggs, and sugar-sweetened beverages would lead to an 80% reduction in T2D risk. 

Our domain scorings fit well into these recommendations, with the exception that in our “grains” domain, to gain maximum points one needs to consume six portions of whole grains daily. This discrepancy may partly originate from the cultural differences in food intake [49], as observational studies can only give a prediction of the risk across the consumption distribution within the included study populations. In Finnish food culture, whole-grain rye bread and oatmeal porridge are staples and the recommended daily consumption of cereal products is six portions (one portion being, e.g., 30 g slice of bread) for women and nine portions for men, with at least half of that being whole-grain [20]. In an early Finnish study [50] (baseline data collection between 1966–1972), the median daily intake of whole grains was, by increasing quarter, 79 g, 136 g, 198 g, and 302 g. Associated relative risks of developing type 2 diabetes during 10-year follow-up were 1, 1.05, 0.52 and 0.65 (*p* = 0.02 for trend). This suggests that the plateau in T2D risk reduction is not reached until the consumption exceeds seven daily portions of whole grains. 

### 4.3. The Consistency of the HDI with Other Diet Quality Scores 

The HDI domain contents are compatible with several other diet quality scores where the focus has been on the whole diet and not a specific food group or nutrient [10,13,14], even though the grouping and specific details vary. All scores include measures for the intake of fruit and vegetables (some also legumes), most also whole grains or fibre, and fat (olive oil, nuts, fatty acid categories), and some also meat, fish and dairy. Consumption of sugar-sweetened beverages is typically included in the scores. 

However, there are also differences. In the HDI we included a broad domain entitled “snacks and treats” which in some scores is named “empty calories” covering the consumption of fast foods, salty and sugary snacks, sweets, and desserts, and beverages that contain either sugar or alcohol. Alcohol was thus treated as a source of excess energy [42]. The scores measuring the adherence to the Mediterranean diet typically grade moderate daily consumption of wine as beneficial [33,51]. The question of whether alcohol should be included as part of a health-promoting diet that prevents T2D and what would be the recommended consumption is controversial and was considered to be out of the scope of the HDI [52,53]. 

Dairy products are important in Finnish food culture and provide protein, calcium, and vitamins, but as they are also an important source of saturated fat, it is essential to pay attention to the fat content of the consumed products. Fat quality is a prevailing problem in the Finnish diet in general, with 97% of men and 94% of women consuming more saturated fat than recommended [54] and the high intake is also reflected in the cholesterol levels among the population [55]. In the Finnish national nutrition guidelines [20] the daily use of low-fat dairy, vegetable oils and vegetable-oil-based margarine is recommended and consumption of sources of saturated fat is limited, and this is reflected also in the HDI scoring. In this regard, the HDI is in line with the Dietary Approach to Stop Hypertension (DASH) scores [56]. 

The most obvious difference between the HDI and other scores is the inclusion of a domain “meal pattern” in the HDI, even though the research evidence to recommend a defined meal pattern is not very strong [25,28,29,30]. Practical experience has, however, shown that maintaining a healthy meal pattern is an issue people struggle within their everyday lives, with problems varying from constant grazing to meal skipping followed by uncontrolled eating later on. Therefore, it was considered important to include meal patterns as part of the HDI.

### 4.4. Comparison of the HDI with Dietary Intakes Evaluated with 7-Day Food Records

The HDI and its domains correlated with the calculated nutrient intakes mostly as would be expected. Plausible associations were seen for energy proportion of fat, saturated fat, carbohydrates, and protein, intakes of fibre, magnesium, iron, and vitamin D, as well as a glycaemic index of diet. The fact that there was no association between the HDI and total energy intake offers further validation for the HDI’s ability to estimate dietary quality regardless of energy needs. The present consensus is that diets over a spectrum of distribution of energy-yielding nutrients can be healthy. Nevertheless, in practice, making changes such as increasing whole grains, fruit and vegetables and decreasing foods with high saturated fat content typically lead to an increase in energy proportion of carbohydrates and a decrease in energy proportion of fat [57]. Many expected associations were detected also between nutrient intakes calculated from 7-day food records and the HDI domain points. For example, fibre intake was associated with “grains”, “fruit and vegetables”, “fish and meat”, and “snacks and treats” domain points. Additionally, saturated fat intake was negatively associated with “fruit and vegetables” and “dairy” domain points. 

Some seemingly contradictory associations were also seen. The HDI associated inversely with the energy proportion of monounsaturated fat, even though the scoring of the “fats” domain aimed to reflect the general consensus that monounsaturated fat is either beneficial or neutral as regards T2D risk [36]. A likely explanation for this is that in the Finnish diet, the most important sources of monounsaturated fat are fatty meat and milk products [54], which in our scoring were valued based on their saturated fat content. 

Furthermore, “meal pattern” in this analysis correlated negatively with energy proportion of protein and positively with saccharose intake, indicating that a better meal pattern would associate with decreased intake of protein and increase in sugar. In the Airline study, the original D2D-FIQ was used and thus for the scoring of the “meal pattern” domain a single question on the number of meals and snacks consumed during a day was available. This question did not differentiate between main meals and snacks. Therefore, the “meal pattern” score in this analysis might actually reflect a snack-based food pattern that has indeed been shown to associate with an increase in sugar intake [41]. In the updated version of the D2D-FIQ used in the StopDia, separate questions were asked about main meals and snacks, to avoid this discrepancy. 

### 4.5. Association of the HDI with Clinical Risk Factors

Age was positively associated with quality of diet in the StopDia data, measured as the HDI. The same phenomenon, suggesting older people having a more healthy diet than younger, has been seen in the Finnish population-based survey [54], and thus probably reflects a true association also in the present dataset. The HDI was inversely associated with BMI, with 1.9 and 1.4 BMI-units difference between the lowest and highest HDI quarter in women and men, respectively; however, causality between diet and obesity cannot be confirmed with this cross-sectional analysis. As impaired glucose and lipid metabolism, in turn, is known to associate both with obesity [58] and age [59], the other analyses on associations between clinical risk factors and the HDI were conducted also adjusting for age and BMI to avoid confounding. 

Statistically significant associations were seen between the HDI and glycaemia indices as well as serum triglycerides. The differences in risk factors between HDI quarters were moderate, but consistent with the aim of the D2D-FIQ to cover the intake of foods that are related to metabolic factors that increase T2D risk. Finally, in this cross-sectional analysis adjusted for BMI and age, each 10-point increase in HDI was associated with a 0.6 cm reduction in waist circumference, and improvements in many other risk factors. Putting this to a scale, increasing the HDI by 30 points (the difference in the means between lowest and highest quarter) by making achievable beneficial changes to diet could reduce the waist circumference by 1.8 cm even without weight reduction. A difference of this magnitude is not insignificant: in the DPS, where a 58% reduction in the incidence of T2D was seen in the intervention group compared with the control group, the reduction in waist circumference during the first year was in average 4.4 cm in the intervention group and 1.3 cm in the control group [47]. 

### 4.6. Justification for the Proposed Use of the HDI

Nutrition has a central role in the prevention and treatment of many common chronic diseases, and the same dietary principles are applicable to the prevention and treatment of T2D, cardiovascular diseases, and obesity [32]. Even though the development of HDI was guided by the evidence on diet in the prevention of T2D, it is general enough to suit for the counselling of other patient groups also. It would support the holistic approach to nutrition: even in diseases with specific dietary requirements, it is important not to overlook the general principles of a healthy diet. Furthermore, the HDI could help setting and monitoring of small, achievable short-term goals, which has been proposed as a strategy to increase the likelihood of forming a permanent habit [12].

Dietary counselling should preferably be delivered by nutrition professionals with specific expertise in nutrition therapy [4], but as registered dietitians are a scarce resource in healthcare in many countries, including Finland [60], counselling is typically conducted by other healthcare professionals, such as physicians and nurses. If the councellors’ training on nutrition is superficial, they may be inclined to focus on single nutrients or foods rather than taking into account the contributions of different food groups in a health-promoting diet. Healthcare workers’ lack of sufficient knowledge, skills, and competence in dietary issues has been identified as one of the barriers against practicing dietary counselling [11,61]. A systematic review comparing nutrition therapy delivered by registered dietitians with dietary advice provided by other healthcare professionals revealed clear differences in the achieved outcomes [62]. A practical and concrete tool such as the HDI could improve the effectiveness and support the continuation and coherence of the counselling provided by alternating professionals.

The D2D-FIQ takes about 15 min to fill out. Brief screeners with fewer questions [63] and even a single question (“In general, how healthy is your overall diet?”) [64] have been proposed as a quick method to identify people with unhealthy diets. Such screeners could indeed be useful for initiating a discussion about diet and lifestyles. However, using the D2D-FIQ as a dietary intake evaluation tool, complemented with automatized calculation and visualization of HDI and its domains could offer a practical approach to support dietary counselling, especially when provided by healthcare professionals with less expertise in nutrition. In their recent scientific statement, the American Heart Association proposes a comparable process for dietary advice in healthcare [11].

### 4.7. Strengths and Limitations

The strength of our approach was that the work was founded on the D2D-FIQ, a questionnaire to evaluate an individual’s food intake that had been created during an earlier T2D prevention program and validated both for accuracy [18] and feasibility [15]. The HDI was developed through a collaboration of nutrition experts and was based on evidence rather than intake distribution within a study population. Furthermore, we were able to validate the HDI against nutrient intake data collected with 7-day food diaries and study its associations with clinical risk factors. The dietary intake in the validation data participants seemed to be comparable to that of the general Finnish population [54] which increases the generalizability of the findings. 

A limitation that needs to be mentioned was that the D2D-FIQ that was used as the basis of the HDI was a slightly updated version from the original, validated one. The additions were minor and presumably did not impair the performance of the D2D-FIQ. It should be noted that the HDI may not be, without adjustments, applicable for exclusion diets such as vegan dietary patterns, which are generally acknowledged as healthy dietary options. Another shortcoming is that the D2D-FIQ did not include a specific question about legume consumption, which has been shown to be associated with reduced T2D risk [65]. The Finnish dietary recommendations endorse legumes, but the consumption is still scarce [54]. Adding a separate question about legume consumption into the D2D-FIQ and including legumes into the calculation of HDI would also be a way to increase knowledge about their benefits in diet.

## 5. Conclusions

In our study, we constructed a Healthy Diet Index (HDI) based on the available short food intake questionnaire D2D-FIQ and explored its criterion validity. The HDI and its domains were shown to correlate with nutrient intakes calculated from 7-day food records. Furthermore, the HDI was associated with clinical and anthropometric risk factors in a cross-sectional dataset. We conclude that the HDI is a valid tool to measure adherence to a health-promoting diet. The HDI can be used to simplify the information from the D2D-FIQ in parameters that allow rapid evaluation of the diet. We propose that using the D2D-FIQ, complemented with automatized calculation and visualisation of the HDI and its domains could be a practical approach to implement dietary counselling in healthcare, especially when other professionals than registered dietitians are delivering the counselling, in order to tackle unhealthy dietary behaviours and risk factors for chronic diseases such as T2D.

## Figures and Tables

**Table 1:** Characteristics (mean ± standard deviation or %) of validation populations.

Characteristic	Airline Dataset		StopDia Dataset	
	Women (*n* = 25)	Men (*n* = 52)	Women (*n* = 2455)	Men (*n* = 645)
Age, years	42.7 ± 8.0	45.0 ± 9.5	54.5 ± 10.1	56.9 ± 9.3
Healthy Diet Index HDI	61 ± 9	55 ± 10	63 ± 11	59 ± 10
Cohabitant, %	68	78	75	85
Education, polytechnic or academic, %	16	12	67	57
BMI, kg/m^2^	26.5 ± 4.4	26.9 ± 3.9	31.3 ± 5.7	31.2 ± 4.8
Total cholesterol, mmol/L	5.0 ± 0.8	5.2 ± 1.0	5.3 ± 0.9	5.1 ± 1.3
Fasting plasma glucose, mmol/L	5.4 ± 0.5	5.5 ± 0.4	5.6 ± 0.7	6.0 ± 0.8
Triglycerides, mmol/L	1.2 ± 0.6	1.4 ± 0.7	1.4 ± 0.7	1.7 ± 1.1
Total energy intake, kJ	6865 ± 966	9310 ± 2006	-	-
Energy proportion of carbohydrates, %	44 ± 8	45 ± 6	-	-
Energy proportion of protein, %	17 ± 3	17 ± 2	-	-
Energy proportion of fat, %	33 ± 7	33 ± 5	-	-
Energy proportion of saturated fat, %	12 ± 4	12 ± 3	-	-
Energy proportion of alcohol, %	5 ± 4	5 ± 4	-	-
Fibre, g/4186 kJ	10 ± 3	11 ± 3	-	-
Saccharose, g	49 ± 29	36 ± 19	-	-

BMI = Body mass index.

**Table 2:** Correlation between Healthy Diet Index (HDI) and its domains and dietary intakes calculated from 7-day food records among 77 men and women (the Airline data).

		HDI	Meal Pattern	Grains	Fruit and Vegetables	Fats	Meat and Fish	Dairy	Snacks and Treats
Energy, KJ	***r*** ***p-*value**	−0.14 0.21	0.15 0.19	0.07 0.54	**−0.32** 0.004	−0.12 0.31	−0.07 0.52	−0.08 0.47	**−0.27** 0.016
Carbohydrate, E%	***r*** ***p-*value**	**0.23** 0.049	0.00 0.99	**0.24** 0.032	0.18 0.12	−0.08 0.49	0.08 0.47	0.01 0.91	−0.10 0.37
Fat, E%	***r*** ***p-*value**	**−0.37** 0.001	0.13 0.27	−0.20 0.08	**−0.27** 0.017	−0.05 0.64	−0.19 0.09	−0.16 0.14	−0.09 0.48
Protein, E%	***r*** ***p-*value**	**0.25** 0.033	**−0.23** 0.045	0.10 0.37	0.07 0.52	**0.22** 0.049	−0.04 0.73	**0.39** <0.001	**0.38** <0.001
Fibre, g per kJ	***r*** ***p-*value**	**0.66** <0.001	−0.17 0.15	**0.43** <0.001	**0.58** <0.001	0.00 0.99	**0.24** 0.034	0.19 0.10	**0.33** 0.004
Saturated fat, E%	***r*** ***p-*value**	**−0.39** <0.001	0.21 0.06	−0.19 0.09	**−0.30** 0.008	−0.16 0.15	−0.17 0.14	**−0.28** 0.013	−0.14 0.23
Mono-unsaturated fat, E%	***r*** ***p-*value**	**−0.37** 0.001	0.06 0.62	−0.17 0.14	**−0.30** 0.008	0.00 0.95	−0.20 0.07	−0.14 0.21	−0.12 0.29
Poly-unsaturated fat, E%	***r*** ***p-*value**	−0.12 0.28	−0.15 0.21	−0.06 0.62	−0.09 0.41	**0.23** 0.037	−0.20 0.07	0.10 0.40	0.06 0.57
Saccharose, g	***r*** ***p-*value**	−0.11 0.36	**0.24** 0.039	0.05 0.68	−0.21 0.07	−0.09 0.41	−0.07 0.53	−0.13 0.26	**−0.28** 0.013
NaCl, mg	***r*** ***p-*value**	−0.08 0.49	−0.04 0.75	0.10 0.40	**−0.29** 0.008	0.02 0.86	−0.11 0.35	−0.03 0.78	−0.09 0.43
Magnesium, mg	***r*** ***p-*value**	**0.26** 0.02	0.01 0.91	**0.26** 0.022	0.06 0.63	−0.14 0.23	0.15 0.19	0.07 0.52	−0.04 0.74
Iron, mg	***r*** ***p-*value**	**0.25** 0.029	0.04 0.74	**0.24** 0.036	0.05 0.66	−0.08 0.50	0.12 0.28	0.08 0.48	0.01 0.91
Vitamin D, ug	***r*** ***p-*value**	**0.27** 0.018	−0.16 0.16	0.11 0.35	0.13 0.25	0.07 0.56	0.17 0.13	0.22 0.06	0.20 0.08
Glycaemic index	***r*** ***p-*value**	**−0.32** 0.006	−0.16 0.17	−0.11 0.32	**−0.25** 0.030	−0.02 0.87	−0.12 0.29	**−0.28** 0.013	−0.07 0.57

*r* = Pearson correlation coefficient; E% = Proportion of energy intake. Statistically significant (*p* < 0.05) correlation coefficients that suggest either acceptable (0.20 ≤ |r| ≤ 0.49) or good (|r| ≥ 0.50) association are marked with bold.

**Table 3:** Association between Healthy Diet Index (HDI) and age, anthropometrics, blood pressure, and biomarkers (crude and age- and BMI-adjusted) among women in StopDia data.

Characteristic	Adjustment	Healthy Diet Index				*p* for Trend
		1st Quarter (11–55)	2nd Quarter (56–63)	3rd Quarter (64–70)	4th Quarter (71–93)	
		Mean ± SD	Mean ± SD	Mean ± SD	Mean ± SD	
Healthy Diet Index, HDI (*n* = 2455)		49 ± 5	60 ± 2	67 ± 2	77 ±5	
Age, years (*n* = 2455)		50.4 ± 10.2	53.8 ± 9.7	56.3 ± 9.7	58.2 ± 9.7	<0.001
**Anthropometrics**						
BMI, kg/m^2^ (*n* = 2455)		32.2 ± 5.8	31.3 ± 5.6	31.3 ± 5.8	30.3 ± 5.5	<0.001
Waist circumference, cm (*n* = 2453)	crude	102.1 ± 13.4	100.3 ± 12.4	100.2 ± 13.2	97.8 ± 13.0	<0.001
	adjusted	100.8	100.3	100.0	99.4	<0.001
**Blood pressure**						
Diastolic blood pressure, mmHg (*n* = 2453)	crude	88.0 ± 10.0	87.6 ± 9.6	87.3 ± 9.5	87.3 ± 9.6	0.17
	adjusted	88.0	87.6	87.1	87.4	0.20
Systolic blood pressure, mmHg (*n* = 2453)	crude	136.9 ± 17.6	136.7 ± 17.4	138.7 ± 17.9	140.0 ± 18.1	<0.001
	adjusted	138.7	137.1	137.8	138.6	0.89
**Blood biomarkers**						
HbA1c, mmol/mol (*n* = 2377)	crude	35.9 ± 5.4	36.2 ± 4.3	36.2 ± 4.0	36.3 ± 4.1	0.10
	adjusted	36.3	36.4	36.0	36.0	0.12
Fasting plasma glucose, mmol/L (*n* = 2389)	crude	5.6 ± 0.9	5.6 ± 0.6	5.6 ± 0.6	5.6 ± 0.6	0.08
	adjusted	5.6	5.6	5.5	5.6	0.014
Plasma glucose 2 h from OGTT, mmol/L (*n* = 2388)	crude	6.7 ± 2.5	6.6 ± 2.1	6.4 ± 2.1	6.4 ± 1.9	0.011
	adjusted	6.7	6.6	6.4	6.4	0.007
Fasting plasma insulin, mU/L (*n* = 2321)	crude	13.0 ± 8.3	12.5 ± 9.6	11.7 ± 7.7	11.0 ± 7.7	<0.001
	adjusted	12.4	12.4	11.8	11.6	0.06
Total cholesterol, mmol/L (*n* = 2378)	crude	5.3 ± 0.9	5.3 ± 1.0	5.3 ± 1.0	5.2 ± 0.9	0.034
	adjusted	5.4	5.3	5.2	5.1	<0.001
LDL cholesterol, mmol/L (*n* = 2378)	crude	3.3 ± 0.9	3.3 ± 0.9	3.2 ± 0.9	3.2 ± 0.8	0.002
	adjusted	3.4	3.3	3.2	3.1	<0.001
HDL cholesterol, mmol/L (*n* = 2378)	crude	1.5 ± 0.4	1.6 ± 0.4	1.6± 0.4	1.6 ± 0.4	<0.001
	adjusted	1.6	1.6	1.6	1.6	0.40
Triglycerides, mmol/L (*n* = 2378)	crude	1.4 ± 0.7	1.3 ± 0.7	1.4 ± 0.6	1.3 ± 0.6	0.030
	adjusted	1.4	1.4	1.4	1.3	0.025

SD = Standard deviation; BMI = Body mass index; HbA1c = Glycated hemoglobin; OGTT = Oral glucose tolerance test; LDL = Low-density lipoprotein; HDL = High-density lipoprotein.

**Table 4:** Association between Healthy Diet Score and age, anthropometrics, blood pressure, and biomarkers (crude and age- and BMI-adjusted) among men in StopDia data.

Characteristic	Adjustment	Healthy Diet Index				*p* for Trend
		1st Quarter (30–51)	2nd Quarter (52–59)	3rd Quarter (60–66)	4th Quarter (67–93)	
		Mean ± SD	Mean ± SD	Mean ± SD	Mean ± SD	
Healthy Diet Index, HDI (*n* = 645)		46 ± 5	56 ± 2	63 ± 2	72 ± 5	
Age, years (*n* = 645)		52.8 ± 10.2	56.0 ± 8.9	58.3 ± 8.0	61.3 ± 7.6	<0.001
**Anthropometrics**						
BMI, kg/m^2^ (*n* = 644)		32.1 ± 4.9	31.3 ± 4.6	30.6 ± 4.8	30.7 ± 4.9	0.003
Waist circumference, cm (*n* = 644)	crude	111.7 ± 12.3	110.0 ± 11.8	108.0 ± 12.3	108.0 ± 11.8	0.002
	adjusted	110.1	109.9	109.2	108.6	0.006
**Blood pressure**						
Diastolic blood pressure, mmHg (*n* = 644)	crude	91.9 ± 10.5	91.1 ± 9.1	90.5 ± 10.6	90.6 ± 11.2	0.21
	adjusted	91.3	91.0	90.8	91.1	0.84
Systolic blood pressure, mmHg (*n* = 644)	crude	146.4 ± 15.3	146.3 ± 16.0	146.7 ± 16.6	147.2 ± 18.7	0.63
	adjusted	146.9	146.4	146.7	146.5	0.89
**Blood biomarkers**						
HbA1c, mmol/mol (*n* = 615)	crude	37.7 ± 5.8	37.6 ± 5.5	37.4 ± 4.9	37.5 ± 4.2	0.71
	adjusted	38.1	37.7	37.4	37.1	0.09
Fasting plasma glucose, mmol/L (*n* = 630)	crude	6.1 ± 0.9	5.9 ± 0.7	5.9 ± 0.7	6.0 ± 0.7	0.13
	adjusted	6.1	5.9	5.9	5.9	0.002
Plasma glucose 2 h from OGTT, mmol/L (*n* = 630)	crude	7.7 ± 3.3	7.0 ± 2.3	6.9 ± 2.2	7.0 ± 2.5	0.012
	adjusted	7.8	7.0	6.9	6.9	0.003
Fasting plasma insulin, mU/L (*n* = 599)	crude	17.5 ± 12.9	17.3 ± 22.2	12.7 ± 7.8	13.6 ± 8.5	0.002
	adjusted	16.6	17.1	13.3	14.1	0.032
Total cholesterol, mmol/L (*n* = 615)	crude	5.3 ± 1.9	5.2 ± 1.0	5.1 ± 0.8	4.8 ± 1.1	0.002
	adjusted	5.2	5.1	5.1	4.9	0.06
LDL cholesterol, mmol/L (*n* = 615)	crude	3.2 ± 0.9	3.3 ± 0.9	3.2 ± 0.8	3.1 ± 1.0	0.17
	adjusted	3.1	3.3	3.2	3.2	0.80
HDL cholesterol, mmol/L (*n* = 615)	crude	1.3 ± 0.3	1.3 ± 0.3	1.3 ± 0.3	1.3 ± 0.4	0.17
	adjusted	1.3	1.3	1.3	1.3	0.71
Triglycerides, mmol/L (*n* = 615)	crude	2.1 ± 1.5	1.7 ± 0.8	1.6 ± 0.9	1.4 ± 0.7	<0.001
	adjusted	2.0	1.7	1.6	1.4	<0.001

SD = Standard deviation; BMI = Body mass index; HbA1c = Glycated hemoglobin; OGTT = Oral glucose tolerance test; LDL = Low-density lipoprotein; HDL = High-density lipoprotein.

## Data Availability

The data used in the study are not publicly available due to restrictions stated in the ethical clearance documents. For more information, please contact the corresponding author, J.L.

## Appendix A

18-Item Food Intake Questionnaire (D2D-FIQ)

Think about your typical diet and answer the following questions about eating and drinking. Depending on the question, choose the most appropriate option or answer by a number.

*1. On how many weekdays do you **usually** eat the following main meals?*

**1. Breakfast**	**2. Lunch**	**3. Dinner**
1. I do not eat 2. 1–2 times a week 3. 3–4 times a week 4. Every weekday	1. I do not eat 2. 1–2 times a week 3. 3–4 times a week 4. Every weekday	1. I do not eat 2. 1–2 times a week 3. 3–4 times a week 4. Every weekday

*2. On how many weekdays do you **usually** eat the following snacks?*

**1. Morning Snack**	**2. Afternoon Snack**	**3. Evening Snack**	**4. Other Snacks**
1. I do not eat 2. 1–2 times a week 3. 3–4 times a week 4. Every weekday	1. I do not eat 2. 1–2 times a week 3. 3–4 times a week 4. Every weekday	1. I do not eat 2. 1–2 times a week 3. 3–4 times a week 4. Every weekday	1. I do not eat 2. 1–2 times a week 3. 3–4 times a week 4. Every weekday

*3. How many servings **a week** do you **usually** eat the following dishes as a main course?*

1. ____ fish dishes (e.g., baked fish, fried Baltic herrings, fish soup, herring, or salmon) a week
2. ____ sausage dishes (e.g., baked sausage, sausage soup, sausage stew) a week
3. ____ poultry dishes (e.g., grilled chicken, chicken fricassee, chicken salad) a week
4. ____ meat dishes (e.g., meat soup, steak, meatballs, pork chop, liver casserole) a week
5. ____ vegetarian dishes (e.g., vegetable soup, spinach pancake, vegetable salad) a week

*4. How often do you **usually** eat fast food ? One serving is, e.g., 1 meat pie, 1 hamburger, 1 slice of pizza or 1 dL of chips, popcorn, or salted nuts.*

1. A serving or more per day
2. 4–6 servings a week
3. 1–3 servings a week
4. 1–3 servings a month
5. Less than 1 serving a month or none

*5. What type of cooking fat or oil is **most often** used in your household?*

1. Vegetable oil or liquid margarine
2. Vegetable margarine with 55–80% fat
3. Plant stanol or sterol margarine
4. Hard cooking margarine
5. Butter-vegetable oil mixture
6. Butter
7. Nothing/we do not cook at home

*6. What type of cream is **most often** used in your household for cooking?*

1. Cream-vegetable oil mixture or plant-based cream (e.g., soy or oat cream)
2. Yoghurt for food preparation
3. Low fat cream, low fat crème fraîche, low fat sour cream, cultured half cream
4. Double cream, crème fraîche or sour cream
5. Nothing/we do not cook at home

*7. How much do you **usually** eat vegetables and roots? One serving is, e.g., 1 dL of grated vegetables, salad or cooked vegetables, 1 medium-sized carrot or 2 tomatoes.*

1. 3 servings or more per day
2. 2 servings per day
3. 1 serving per day
4. 4–6 servings a week
5. 1–3 servings a week
6. Less than 1 serving a week or none

*8. How much do you **usually** eat fruit and berries? One serving is a medium-sized fruit or 1 dL of fresh berries.*

1. 2 servings or more per day
2. 1 serving per day
3. 4–6 servings a week
4. 1–3 servings a week
5. Less than 1 serving a week or none

*9. How much do you **usually** eat nuts, seeds, and almonds? One serving is 30 g or 2 tablespoons.*

1. 2 servings or more per day
2. 1 serving per day
3. 4–6 servings a week
4. 1–3 servings a week
5. Less than 1 serving a week or none

*10. What kind of salad dressing do you **usually** use?*

1. I do not usually use salad dressing
2. Vegetable oil (e.g., olive, rapeseed, or linseed oil) or oil-based dressing (e.g., French dressing or mayonnaise)
3. Juice-based dressing
4. Cultured half cream- or yogurt-based dressing

*11. How many decilitres of milk or liquid milk products do you **usually** consume **per day** (milk, buttermilk, yogurt, fermented milk product, curd)? 1 glass = 2 dL.*

1. ____ dL of milk products with <1% fat (e.g., skimmed milk or fat-free yogurt) per day
2. ____ dL of milk products with <2% fat (e.g., semi-skimmed milk) per day
3. ____ dL of milk products with <3% fat (e.g., full fat milk or regular yogurt) per day
4. ____ dL of milk products with >3% fat (e.g., butter milk or yogurt, whole milk) per day

*12. How much bread and other cereals do you **usually** eat **per day**? A slice is a ready cut slice or half of a roll.*

1. ____ slice(s) of rye- or crispbread per day
2. ____ slice(s) of graham-, oat- or mixed grain bread or roll per day
3. ____ slice(s) of white bread or baguette per day
4. ____ dL of porridge (e.g., oat-, rye- or wheat flake porridge) per day
5. ____ dL of low-fibre breakfast cereals (e.g., corn flakes or rice krispies) per day
6. ____ dL of muesli per day
7. ____ slice(s) of sweet bun per day

*13. What kind of spread do you **usually** use on your bread?*

1. Margarine with 30–40% fat
2. Margarine with 55–80% fat
3. Plant stanol or sterol margarine
4. Butter-vegetable oil mixture
5. Butter
6. I do not usually have fat spread on bread

*14. How much cheese do you **usually** eat **per day**? A slice of cheese is about 10 g.*

1. ____ slice(s) of cheese with 17% fat or less per day
2. ____ slice(s) of cheese with >17% fat per day
3. ____ slice(s) of vegetable fat-based cheese per day

*15. How much cold cuts do you **usually** eat **per day**?*

1. ____ slice(s) of cold cuts per day
2. ____ slice(s) of sausages per day
3. ____ piece(s) of frankfurters or barbeque sausages per day

*16. How much sweet pastries, ice cream, puddings or chocolate do you **usually** eat? One serving is, e.g., a piece of pie or cake, a small Danish pastry or doughnut, 3–4 cookies, an ice cream cornet, pudding, or chocolate bar.*

1. 2 servings or more per day
2. 1 serving per day
3. 4–6 servings a week
4. 1–3 servings a week
5. Less than 1 serving a week or none

*17. How much sugar, honey or sweets do you **usually** eat? One serving is, e.g., 2 teaspoons of sugar or honey, 3 sugar lumps, 5 sweets or half of a pastille pack.*

1. 2 servings or more per day
2. 1 serving per day
3. 4–6 servings a week
4. 1–3 servings a week
5. Less than 1 serving week or none

*18. How much do you usually drink the following beverages on average **a week**? If you do not drink any of the following beverages or drink less than one serving a week, mark 0 for that beverage.*

1. ____ cup(s) of tea (1 cup = 2 dL) a week
2. ____ cup(s) of coffee (1 cup = 1 dL) a week
3. ____ glass(es) of water (1 glass = 2 dL) a week
4. ____ bottle(s) of soft drinks or energy drinks with sugar (1 bottle = 1/3 L) a week
5. ____ bottle(s) of sugar-free soft drinks or energy drinks (e.g., light cola, 1 bottle = 1/3 L) a week
6. ____ glass(es) of fruit juice (1 glass = 2 dL) a week
7. ____ glass(es) of sugar-sweetened juice (1 glass = 2 dL) a week
8. ____ bottle(s) of beer, cider etc. with alcohol 4.7% or less (1 bottle = 1/3 L) a week
9. ____ bottle(s) of beer, cider etc. with alcohol over 4.7% (1 bottle = 1/3 L) a week
10. ____ glass(es) of wine (1 glass = 12 cL) a week
11. ____ servings(s) of spirits (e.g., vodka, whisky, cognac, etc., 1 portion = 4 cL) a week

________________________________________________________________________________________________

## Appendix B

**Table A1:** Healthy Diet Index (HDI) justification and scoring based on D2D Food intake questionnaire (D2D-FIQ).

HDI Domain and Justification for Scoring	Maximum Points	Related Question(s) in the D2D-FIQ and the Scoring Principles
**Meal pattern** *		
Nutrition recommendations: regular meal rhythm, having snacks when needed but avoiding grazing; having healthy and balanced meals Research evidence [25]	10 points	Consumption of meals (Q1) and/or snacks (Q2) on weekdays (Mon-Fri): ◦ breakfast or morning snack: every day = 2 points/on 2–4 days = 1 point/on 0–1 days = 0 points ◦ lunch or afternoon snack: every day = 2 points/on 2–4 days = 1 point/on 0–1 days = 0 points ◦ dinner or evening snack: every day = 2 points/on 2–4 days = 1 point/on 0–1 days = 0 points ◦ number of meals and snacks combined: 4–6 per day = 1 point/<4 or >6 per day = 0 points Number of main course dishes consumed per week (Q3): ≥7 dishes = 3 points/4–6 dishes = 2 points/1–3 dishes = 1 point/0 dishes = 0 points
2. **Grains**		
Nutrition recommendations: favour whole-grain, high-fibre cereals, circa 6 servings of grain products per day Research evidence [21,22,23,35]	20 points	Consumption of grain foods slices/dL per day (Q12): ◦ weighed sum based on fibre content: (1 x rye bread + 0.5 x graham bread + 0.5 x porridge + 0.8 x muesli): 6 or more = 17 points/5–<6 = 15 points/4–<5 = 12 points/3–<4 = 9 points/2–<3 = 6 points/1–<2 = 3 points/0–<1 = 0 points Sum of low-fibre grain foods slices/dL per day: (white bread + cereals + sweet bun): ≤1 per day = 3 points/>1 per day = 0 points
3. **Fruit and vegetables**		
Nutrition recommendations: at least 250 g of vegetables and roots (≥3 servings) and 250 g of fruit and berries per day (≥2 servings) per day Research evidence [23,33]	20 points	Consumption of vegetables (Q7): ≥3 servings per day = 12 points/2 servings per day = 8 points/1 serving per day = 4 points/4–6 servings per week = 2 points/1–3 servings per week = 1 point/<1 serving a week = 0 points. Consumption of fruit and berries (Q8) per day: ≥2 servings per day = 8 points/1 serving per day = 5 points/4–6 servings per week = 2 points/1–3 servings per week = 1 point/<1 serving a week = 0
4. **Fats** **		
Nutrition recommendations: favour vegetable oils, vegetable oil-based fat spreads and dressings, nuts and seeds, avoid butter and fats high in saturated fat; nuts, seeds and almonds circa 30 g per dayResearch evidence [35,36]	15 points	Cooking fat (Q5): Vegetable oil, liquid margarine, margarine with 55–80% fat, plant sterol or stanol margarine = 2 points/hard cooking margarine, butter-vegetable oil mixture, nothing or do not prepare food at home = 1 point/butter = 0 points Cream in cooking (Q6): Cream–vegetable oil mixture, soy or oat cream, nothing or does not prepare food at home = 2 points/reduced fat cream, crème fraîche, sour cream, cooking yogurt, or cultured half cream = 1 point/full-fat cream, crème fraîche, sour cream = 0 points Salad dressing (Q10): Vegetable oil or oil-based dressing = 3 points/juice-based dressing, cultured half cream- or yogurt-based dressing, or does not use = 0 points Spread on bread (Q13): Margarine with 55–80% fat or plant stanol or sterol margarine = 6 points/margarine with 30–40% fat = 2 points/butter-vegetable oil mixture = 1 point/butter or does not use = 0 points. Consumption of nuts, seeds, and almonds (Q9): 1 serving or more per day = 2 points/1–6 servings per week = 1 point/less than 1 serving per week = 0 points
5. **Fish and meat**		
Nutrition recommendation: ≤500 g of cooked red meat or processed meat products per week (≥4 servings a week); fish ≥2 times per week Research evidence [23,24]	10 points	Consumption of dishes per week (Q3): ◦ fish: ≥2 dishes = 2 points/<2 dishes = 0 points ◦ sausage: <1 dishes = 1 point/≥1 dish = 0 points ◦ meat: ≤3 dishes = 1 point/>3 dishes = 0 points ◦ vegetarian: ≥2 dishes = 1 point/<2 dishes = 0 points Consumption of processed meats per day (Q15): ◦ cold cuts: 0–2 slice(s) = 2 points/3–4 slices = 1 point/>4 slices = 0 points ◦ cold cut sausages: 0 slice = 2 points/1 slice = 1 point/>1 slice = 0 points ◦ frankfurters: 0 piece = 1 point/>0 piece = 0 points
6. **Dairy**		
Nutrition recommendations: 5–6 dL of low-fat liquid milk products per day; 2–3 slices of cheese with ≤17% fat or vegetable fat-based cheese per day Research evidence [23,27,38]	10 points	Consumption of liquid milk products per day (Q11): ◦ milk products with <2% fat: 5–6 dL per day = 3 points/1–<5 dL or 6– <10 dL = 2 points/<1 dL or ≥10 dL = 0 points ◦ milk products with 2 to <3% fat: 0–2 dL = 1 point/>2 dL = 0 points ◦ milk products with >3% fat: 0 dL = 1 point/>0 dL = 0 points Consumption of cheese per day (Q14): ◦ cheese with ≤17% fat or vegetable fat-based cheese: 2–3 slices = 3 points/0–1 or 4–6 slices = 1 point/≥7 slices = 0 points ◦ cheese with >17% fat: 0 slices per day = 2 points/1–2 slices = 1 point/>2 slices = 0 points
7. **Snacks and treats**		
Nutrition recommendations: limit intake of salty and sugary snacks and treats, not consumed daily; limit intake of sugary beverages; ≤1 glass of fruit juice per day; limit intake of alcoholic beverages ≤1 serving per day Research evidence [23,26,40]	15 points	Consumption of fast food (Q4): <1 serving per month = 3 points/1–3 servings per month = 2 points/1–3 servings per week = 1 point/≥4 servings per week = 0 points Consumption of sweet pastry, desserts, ice cream and chocolate per week (Q16): <1 serving = 3 points/1–3 servings = 2 points/4–6 servings a week = 1 point/≥1 serving per day = 0 points Consumption of sugar, honey, or sweets (Q17): ≤3 servings per week = 3 points/4–6 servings per week = 2 points/1 serving per day = 1 point/≥2 servings per day = 0 points Consumption of beverages per week (Q18): ◦ sugary beverages (soft drinks with sugar + sugar-sweetened juice): 0 glass = 3 points/>0–2 glasses = 1 point/>2 glasses = 0 points ◦ consumption of fruit juice: ≤7 glasses = 1 point/>7 glasses = 0 points ◦ alcoholic beverages: 0–2 servings = 3 points/>2–4 servings = 2 points/>4–7 servings = 1 point/>7 servings = 0 points

* In the airline validation study, the original FIQ lacking the question on snacks consumption was used and the calculation of the “meal pattern” domain was based on a combined question on number of meals and snacks habitually consumed during a day (1–2 = 1 point/3–4 = 5 points/5–6 = 7 points/≥7 = 2 points) and a question about the number of main courses consumed per week (1–3 = 1 point/4–6 = 2 points/≥7 = 3 points). ** In the airline validation study, the original FIQ lacking the question on the consumption of nuts, seeds, and almonds was used, and therefore the maximum for domain “fats” was 13 points and consequently, the maximum HDI was 98 points.
